# Incidence of postoperative complications in patellar fractures related to different methods of osteosynthesis procedures - a retrospective cohort study

**DOI:** 10.1186/s12891-023-06998-3

**Published:** 2023-11-09

**Authors:** M. V. Neumann –Langen, V. Sontheimer, J. Näscher, K. Izadpanah, H. Schmal, E. J. Kubosch

**Affiliations:** 1 Department of Orthopaedic and Trauma Surgery, Klinikum Konstanz, Mainaustrasse 35, 78464 Konstanz, Germany; 2 Department of Orthopedics and Trauma Surgery, Medical Center – Albert-Ludwigs-University of Freiburg, Hugstetter Strasse 55, 79106 Freiburg, Germany; 3 Department of Mathematics and Statistics, University of Konstanz, Eggerthaldestrasse D, 78457 Konstanz, Germany; 4 Department of Orthopedic Surgery, University Hospital Odense, Sdr. Boulevard 29, 5000 Odense C, Denmark

**Keywords:** Patella, Fracture, Osteosynthesis, Complications, Osteoarthritis

## Abstract

**Background:**

Patellar fractures have a comparatively low incidence compared to all fracture frequencies of the musculoskeletal system. However, surgical management is crucial to prevent postoperative complications that affect the knee joint. The purpose of the present study was to evaluate the incidence of postoperative complications and onset of postoperative osteoarthritis related to the chosen technique of patellar fracture management.

**Methods:**

In a retrospective cohort study consecutive managed, isolated patella fractures were reviewed for demographic data, trauma mechanism, patella fracture type, fixation technique and postoperative complications. The results were documented radiographically and clinically and analysed statistically. The reporting followed the STROBE guidelines.

**Results:**

A total of 112 patients were eligible for data evaluation. Surgical management of comminuted patellar fractures with small fragment screws showed significant fewer postoperative complications compared to other fixation techniques (8%, *p* < 0.043). The incidence of posttraumatic infection was significantly higher following the hybrid fixation technique with cannulated screws and tension wire than following the other analysed techniques (*p* = 0.024). No postoperative wound infection was observed after screw fixation or locking plate fixation. Symptomatic hardware was most frequently seen after tension-band fixation. Onset of posttraumatic osteoarthritis was most often found after the hybrid fixation technique (55%).

**Conclusion:**

Surgical management of patellar fractures remains crucial but fracture fixation using plating systems or small fragment screws is least associated with postoperative complications.

**Trial registration:**

Trial registration number (DRKS):00027894.

## Background

Musculoskeletal injuries related to the bony parts of the knee joints are comparatively rare and based on published data, patellar fractures represent approximately 1 – 3% of all musculoskeletal injuries [1–3]. Data from Scotland report an overall incidence of 9.5/100,000 patella fractures per year [4]. A recent study with a larger population reported an increasing incidence of 13.1/100,000 patella fractures per year with year-to-year variation and a distribution of incidence with increasing age [2].

The increase in patellar fractures follow the more active lifestyle in the younger and older generations leading to more injuries of the musculoskeletal system. Another contributing factor for the increasing number of patellar fractures is periprosthetic patella fractures, which account for up to 2.5% of all patella fractures [5, 6] and implant-associated fractures following e.g. reconstruction of the medial patellofemoral ligament [7, 8]. The patella’s significance as a stabilizer during the extension mechanism and articular congruency of the knee joint is relevant. Hence, surgical restoration of the articular surface is necessary to prevent high contact forces in the patellofemoral joint and to prevent posttraumatic osteoarthritis.

Numerous studies of retrospective analysis and biomechanical implant testing of patellar fractures have been presented. However, few studies have focused on the relationship between the incidence of postoperative complications and the type of osteosynthesis.

The objective of this study was the retrospective analysis of a large patient cohort managed surgically for isolated patellar fractures in trauma centre level I over a decade. The results will provide an overview of postoperative complications after surgical management of isolated patellar fractures, onset of posttraumatic degenerative joint changes and their incidence related to the osteosynthesis procedure. We hypothesized that conventional procedures such as tension band osteosynthesis will have the most postoperative complications compared to modern osteosynthesis procedures such as plate systems or minimally invasive techniques.

## Methods

The clinical database of a Trauma Centre Level I was searched for patients managed for isolated patellar fractures between January 1^st^ 2010 and December 31^st^ 2020 using the International Classification of Diseases (ICD), 10^th^ Revision variable S82.0. Inclusion criteria for the retrospective cohort study with clinical and radiographic analysis were complete patient charts, patient age > 18 years at the time of injury, a patella fracture with indication for surgical fixation and a minimum follow-up of 12 months. The limited period of 12 months was chosen to allow evaluation of late complications such as postoperative late or nonunion or signs of posttraumatic onset or progression of degenerative joint changes.

Exclusion criteria were multiple injured patients, additional knee injury (e.g., knee dislocation with ligamentous or osteochondral injury, distal femoral fracture), juvenile fractures (e.g., sleeve fracture), periprosthetic knee fracture, pathological fracture, and incomplete patient chart. The data were reviewed for fracture classification following the AO-ASIF classification [9] and surgical methods of fracture fixation such as the tension-band wiring technique, small fragment screw fixation, hybrid technique with cannulated screw and tension wire or plate osteosynthesis. Preoperative radiographs were counted as complete with antero-posterior and lateral conventional radiographs, and a missing additional CT scan was not an exclusion criterion.

Radiographic outcome respected the occurrence of degenerative changes postoperatively according to the Kellgren and Lawrence classification [10]. Two independent surgeons reviewed the radiographs for signs of degenerative changes in the knee joint with a focus on the patellofemoral joint, as described by Kellgren and Lawrence. It was documented if there were primary signs of posttraumatic degenerative joint changes, e.g., osteophytes or sclerosis, or progression of preexisting osteoarthritis. The presence of signs of osteoarthritis was observed the earliest 12 months after patellar fracture fixation.

Postoperative complications were considered early if they occurred within 14 days after surgery (wound infection, postoperative haematoma, thromboembolism etc.). Late postoperative complications were recorded with secondary fracture displacement and fracture nonunion.

**Fig. 1:**
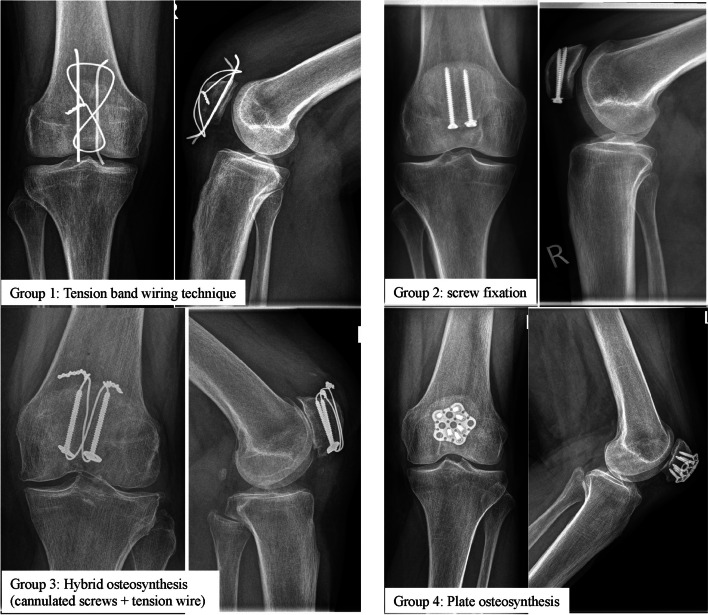
Radiographic examples of surgical managed patellar fractures for each follow-up group

The study cohort was divided into groups according to the technique of osteosynthesis: tension band wiring technique (group 1), screw fixation (group 2), hybrid osteosynthesis with cannulated screw and tension wire (group 3) and plate osteosynthesis (group 4) (Fig. 1).

The postoperative follow-up treatment was partial weight bearing for 6 weeks postoperatively with continued weight-adapted thrombosis prophylaxis in all groups. Patients in groups 1 and 2 had limited flexion of 0–0-30° within the first two weeks following surgery and a two-week increase to 0–0-60° resp. 0–0-90° until 6 weeks postoperatively. Patients in groups 3 and 4 had no flexion limits postoperatively. Splints or braces were not part of the postoperative period after care.

Statistical analyses were performed using R Studio Version 1.2.5019, Posit PBC, Boston, MA, USA. Data were compared using the Fisher’s exact test and post-hoc tests were performed using pairwise Fisher’s exact test with the Benjamini–Hochberg FDR Method at a 5% cut-off. Visualizations were created using „mosaic plot” and „box plot “ in R Studio. Logistic regression was used to examine epidemiological and classification effects. A *p*-value < 0.05 was considered to indicate statistical significance.

The study protocol was approved by the local Human Research Ethics Committee (EK-FRBG-189/17).

## Results

**Fig. 2:**
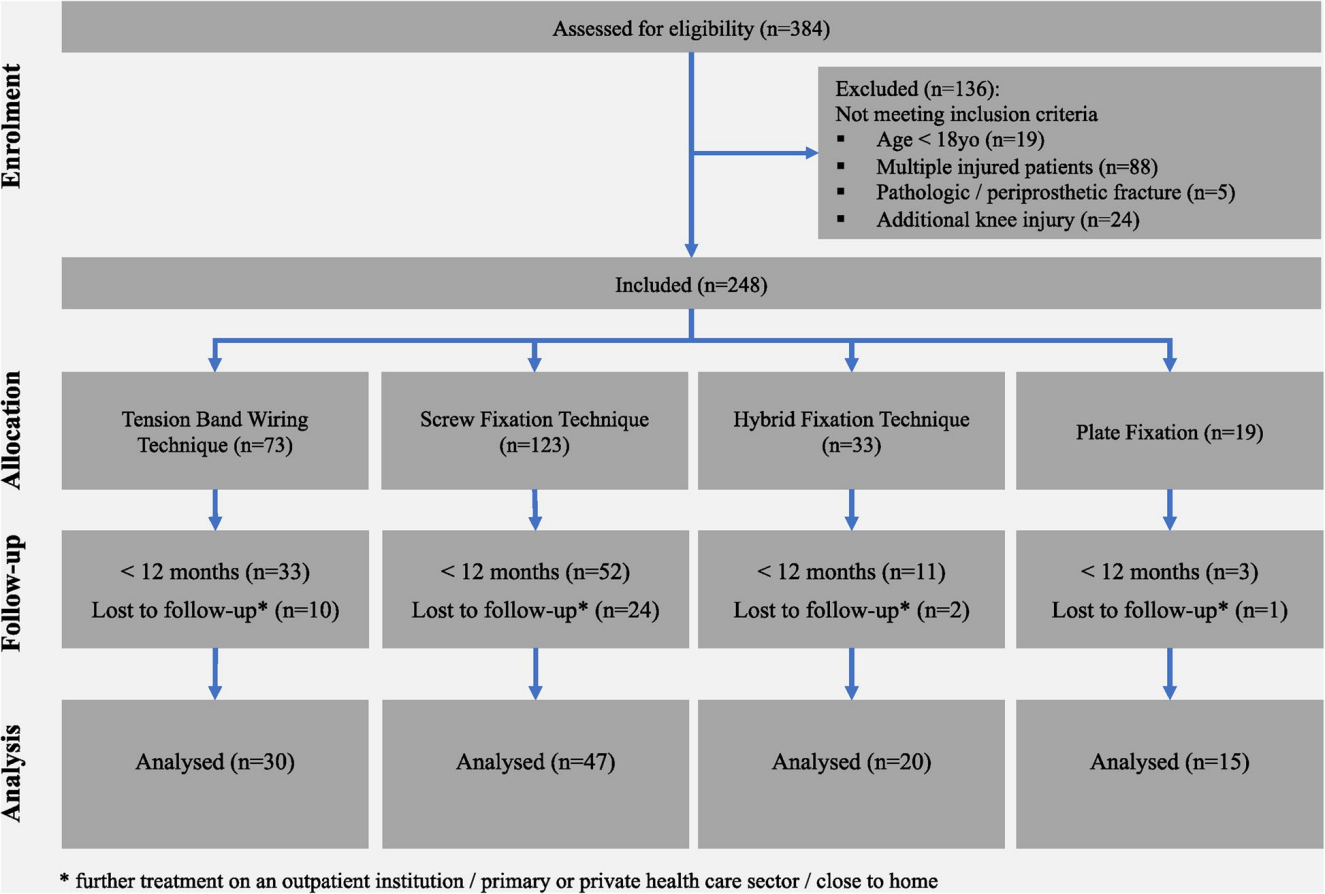
Flow diagram depicting exclusion criteria for the retrospective cohort study

The analysis of the database showed 384 patients with an isolated patella fracture managed surgically at our department within the named period. A total of 136 patients, who did not meet the inclusion criteria were excluded, whereas 248 patients who received surgical management were included. Due to loss to follow-up or follow-up of less than 12 months another 136 patients were excluded. Considering the mentioned inclusion criteria 112 patients remained for analysis (Fig. 2).

The mean follow-up was 17 months (range 12 – 168 months). There were 52 female and 60 male patients reviewed. The mean age at the time of surgical treatment was 48.5 years (range 18–91 years). The trauma incidence of patellar fracture was bimodal with two peaks in the mid-twenties and mid-sixties. Equivalent was the bimodal course of the trauma mechanism with high-energy trauma in young patients, such as traffic and sport injuries and low-energy trauma in elderly patients, such as falls from height or stumble falls.

Mainly comminuted patella fractures of AO type 34C1-3 were managed surgically (*n* = 94), followed by AO type B1-2 as vertical medial or lateral in 18 cases.

The mean age in group 1 was 45 years (18 – 84 years), 47 years (18 – 84 years) in group 2, 47 years (18 – 91 years) in group 3, and 54 years (26 – 72 years) in group 4. The gender distribution was balanced within the groups. The descriptive results including fracture classification and postoperative complications are shown in Table 1.

**Table 1:** Tabular summary of demographic data, fracture classification and postoperative complications

	**Group 1 (tension band technique)**	**Group 2 (screw fixation)**	**Group 3 (hybrid technique)**	**Group 4 (plate osteosynthesis)**
Total number (n)	30	47	20	15
Gender	f = 12, m = 18	f = 22, m = 25	f = 10, m = 10	f = 8, m = 7
Age (years)	45 (18–84)	47 (18–84)	47 (18–91)	54 (26–72)
Follow-up (months)	30 (12–91)	31 (12–168)	24 (12–105)	19 (12–28)
Fracture classification				
AO 34 B1	0	9	0	0
AO 34 B2	0	7	1	1
AO 34 C1	8	23	3	2
AO 34 C2	12	8	5	2
AO 34 C3	10	0	11	10
Postoperative complications				
Early complications (wound infection, postoperative hematoma, thrombembolism	5	2	4	0
Late complications (secondary fracture dislocation, nonunion, osteoarthritis	4	2	1	2
Complications ratio (%)	30%	8.51%	25%	13.33%
Postoperative infections	2	0	3	0
Infections ratio (%)	6.67%	0%	15%	0%
Signs of postraumatic degenerative joint changes	16	18	11	7
Implant removal	23	26	13	11

Younger patients were predominantly managed with the tension-band wiring technique, whereas elderly patients were treated with nonlocking or locking plate systems. Complex AO 34C3 type fractures were mainly fixed with plating systems. The incidence of posttraumatic infection was significantly higher following the hybrid fixation technique (*p* = 0.024). Implant removals were most frequently needed after the tension-band wiring technique and plate osteosynthesis.

**Fig. 3:**
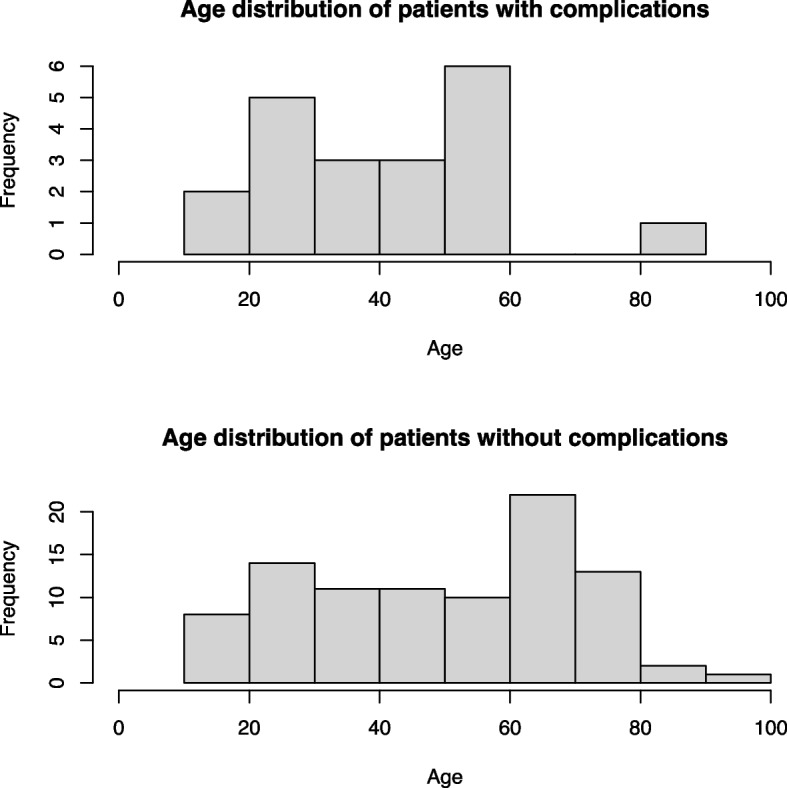
Distribution of affected patients with and without postoperative complications

The age distribution of patients affected with postoperative complications peaked at a mean age of 59 years, and patients who showed no postoperative complications peaked at the age of 66 years (Fig. 3).

Postoperative complications were significantly rarer following small fragment screw fixation compared to all other fixation systems (*p* = 0.043). Direct comparison between group 1 and group 2 showed a significantly lower occurrence of postoperative complications following small fragment screw fixation (group 2) (*p* = 0.026). The direct comparison between the included groups showed no statistical significance for postoperative complications (Table 2).

**Table 2:** Comparison between groups for statistical analysis of postoperative complication rates

Type of Osteosynthesis	Type of Osteosynthesis	*n* = total of analysed patients	p (pairwise fisher-test)
Tension band wiring (*n* = 30)	Hybrid Osteosynthesis (*n* = 20)	50	0.758
Tension band wiring (*n* = 30)	Screw fixation (*n* = 47)	77	0.157
Tension band wiring (*n* = 30)	Plate osteosynthesis (*n* = 15)	45	0.576
Hybrid osteosynthesis (*n* = 20)	Screw fixation (*n* = 47)	67	0.339
Hybrid osteosynthesis (*n* = 20)	Plate osteosynthesis (*n* = 15)	35	0.758
Screw fixation (*n* = 47)	Plate osteosynthesis (*n* = 15)	62	0.758

The refracture rate after open reduction and internal fixation was observed once in group 2, and loss of reduction and secondary dislocation were found in groups 1 and 4.

The results of the first signs of posttraumatic degenerative joint changes or progression of preexisting osteoarthritis according to the Kellgren-Lawrence classification are described in Table 3.

**Table 3:** Overview of radiographic findings of posttraumatic degenerative joint changes or progression of preexisting osteoarthritis

	Group 1	Group 2	Group 3	Group 4
Onset of posttraumatic degenerative joint changes in months (range)	*n* = 13 38.5 (12–91)	*n* = 12 57 (12–168)	*n* = 9 28.4 (12–105)	*n* = 4 23 (17–28)
Progression of pre-existent osteoarthritis in months (range)	*n* = 3 38.3 (12–115)	*n* = 6 37.8 (12–107)	*n* = 2 37 (22, 52)	*n* = 2 18.5 (13, 24)
Total in months (range)	40.125 (12–115)	47.4 (12–168)	32.7 (12–105)	20.75 (13–28)

The investigation revealed that neither age nor gender had any influence on the regression (*p* < 0.001). The regression analysis to other variables showed no significant differences between groups due to inhomogeneity in the numbers of the groups or fewer differences between groups.

## Discussion

The incidence of postoperative complications after various fixation techniques for patellar fractures in this study are as follows: early and late postoperative complications were most often found after the cerclage wiring technique due to less biomechanical stability and fragment compression forces; the first signs of posttraumatic degenerative changes in plain radiographs after the hybrid fixation technique with cannulated screws and tension wire were potentially caused by intraoperative reduction manoeuvres and eventually incongruent fracture reduction. Implant removal was most frequently needed after techniques with osteosynthesis material that was placed and fixed extraosseous, such as tension band wiring technique and plating systems.

There was a tendency for younger patients to have more complications than elderly patients (Fig. 3). This might be due to a more active lifestyle with higher functional demands, eventually minor compliance, and higher numbers of used tension-band wiring techniques. Although locking plate systems have significantly better mechanical outcomes in managing comminuted patella fractures [11, 12], it is often an economic consideration whether to use a locking plate or rather choose the less expensive system of tension band wiring systems.

In the present study it was revealed that small fragment screw fixation was chosen in less comminuted fracture types compared to locking plate systems which were mainly used in comminuted fracture types of AO type 34C3 (Table 1). AO type B fractures are suitable and convenient to treat with screw fixation, in AO type C fractures singular screw fixation is demanding and achieves less stable fracture fixation. This finding mirrors clinical and biomechanical results as locking plate systems allow superior mechanical stability for the reduction of comminuted patellar fractures compared to screw fixation techniques [13]. Angle-stable plate osteosynthesis is currently the most modern osteosynthesis method for patellar fractures. Plate osteosynthesis shows high mechanical stability, which is significantly superior to that of the tension band [11, 14]. The further development of plate systems rely on small screw diameters and the availability of a claw version to address pole fractures. Even if the soft tissues are less compromised than the tension band many patients ask for implant removal [15, 16], which is in accordance with our findings.

Classic tension band osteosynthesis is still the workhorse in the treatment of patellar fractures [17]. The tension band is a technically demanding form of osteosynthesis with a comparatively high complication rate. The assumption that these tensile forces can be converted into compressive forces was refuted not least by Zderic et al [18]. Torchia and Lewallen published in 1996 that 36% needed removal of painful hardware [19]. LeBrun et al. reported symptomatic hardware after patellar fracture fixation with tension band fixation in 52% of patients [20], and Smith et al. specified the requirement of implant removal after the tension-band wiring technique at an average of 7 months postoperatively in 18% of patients [21]. Other authors report irritating hardware ranging from up to 60% [22] with necessary implant removal. In our series implant removal was needed in 76% (*n* = 23/30) following tension band wiring.

Postoperative fixation failure is rarely described. Contributing factors for fixation failure are technical error, fracture comminution or patient noncompliance. Smith et al. reported fixation failures in 22% of a series of 49 cases fixed with the tension band wiring technique [21]. Hoshino et al. reported 4.7% fixation failures in a retrospective study [23]. A retrospective cohort study published in 2021 showed that fixation failure was significant between groups with failure rates of 4.7% after Kirschner wire fixation and 14.5% with cannulated screws [24]. In our series fracture fixation failure was found in 8% of patients mainly following the tension band wiring technique (13%) and hybrid osteosynthesis (20%).

Postoperative infection after surgical management of patellar fractures is reported in 2–10% of cases [21, 23], whereas higher rates of infection are found with increasing damage to the soft tissue [19]. In our reported study the postoperative infection rate was 9.8% which is in accordance with the reported studies.

The occurrence of posttraumatic osteoarthritis is rarely reported. Posttraumatic arthritis develops secondary to a traumatic articular injury, residual joint incongruency, iatrogenic intraarticular injuries or malpositioned hardware [25]. In 1964 Sorensen reported 70% cases of osteoarthritis after patellar fracture fixation, and Mehdi et al. in 1999 published 8.5% of posttraumatic arthritis reviewed on conventional radiographs [26, 27]. In the present cohort study degenerative joint changes in the patellofemoral joint or progression of preexisting osteoarthritis in plain radiographs were first observed after a mean of 36 months following patellar fracture fixation. In all reviewed groups the first signs of degenerative joint changes were found more frequently than signs of progression of preexisting osteoarthritis (Table 2). These radiographic degenerative joint changes according to the Kellgren-Lawrence classification were most frequently found following the hybrid fixation technique with 46% after a mean follow-up of 33 months (12–105 months). The Kellgren-Lawrence classification is used to classify the severity of osteoarthritis on conventional radiographs. However, before radiographic findings of posttraumatic osteoarthritis intraarticular changes define the onset. In addition to anatomical fracture reduction, an imbalance of the joint haemostasis and a disturbed synovial fluid environment with increasing numbers of inflammatory cytokines rule out whether a joint will be affected by further degenerative changes [28, 29].

A recent meta-analysis published in 2011 with a total of 24 studies of patellar fractures reviewed showed that there is a frequency of postoperative infection of 3.2% in 18 studies with a total of 522 fractures [30]. The frequency of nonunion was 1.3% in 15 studies with a total of 464 fractures reviewed. The frequency of reoperation with hardware removal was 33.6%. There are no significant predictors for reoperation, nonunion or infection, but symptomatic hardware leads to a high number of elective hardware removals [30].

The intention of the present study was to evaluate complications following different fixation methods for surgically managed patellar fractures. Although the fracture entity often determines the fixation method, the type of fracture itself is not necessarily related to the occurrence of postoperative complications. Although there are overlaps in the form of treatment, multifragmentary patellar fractures are most often treated with a tension band fixation technique, hybrid procedure or plate osteosynthesis. However, it could be demonstrated that plate osteosynthesis leads to fewer postoperative complications than the hybrid procedure or tension band fixation. Less complex fracture entities were managed predominantly with screw fixation followed by the tension band wiring technique whereas the screw fixation method showed significantly fewer postoperative complications than the tension band wiring technique.

There are some limitations of this study. First, two-thirds of treated patella fractures at our institution were excluded mainly due to a short follow-up period, as well as concomitant injuries to the knee joint. This resulted in a relatively small number of included patients and thus smaller comparison groups. Hence, the statistical significance of postoperative complications between groups was limited and a multivariate analysis could not be carried out. Second, the groups size is unbalanced, therefore statistical outcome is critical to discuss and allows rather a clinical relevance then a statistical relevance. In addition, this was a retrospective cohort study that affected the precision of the data, and it was a single-centre study that limited the achievable data as well. Another limitation that must be considered is that the treatment groups presented different kinds of fractures. This affects both indications as simple fractures are predominantly fixed with tension band wiring and comminuted fractures with hybrid techniques or plate fixation. The data regarding the first signs of posttraumatic degenerative joint changes in plain radiographs is critical to discuss as osteoarthritis follows a dynamic progression with metabolic joint fluid changes, hence its radiographic and temporal significance is limited. The earlier onset of posttraumatic degenerative changes seen following plate and screw fixation is independent of the fixation technique but to the comminution of the fracture itself.

Despite the small size of the comparison groups, the presented results are in accordance with previous studies. The incidence of early and late postoperative complications following surgical management of isolated patellar fractures was 18% in this study. Open reduction and internal fixation using small fragment screws had significantly fewer postoperative complications than other common fixation methods. The incidence of postoperative infection and first signs of radiographic degenerative joint changes was highest following hybrid osteosynthesis. Symptomatic hardware is most frequently seen after tension-band fixation. Locking or nonlocking plating systems and small fragment screw fixation have the lowest incidences of postoperative complications after patellar fracture repair. Small fragment screw fixation is recommended in less comminuted patellar fracture patterns such as vertical or transverse fracture types or solid pole breaks. Modern plating systems seem to be superior in reduction and fixation of comminuted patellar fractures with fewer postoperative complications to expect.

## Conclusions

In summary, surgical treatment of patellar fractures is associated with a relatively high complication rate overall. As expected in intraarticular fractures, degenerative changes are frequently seen in plain radiographs only a few years after injury. Further long-term and multicentre studies including patient-reported outcome measurements and functional outcomes are necessary to elicit the best stabilization technique for different patella fracture patterns.

## Data Availability

All data generated or analysed during this study are included in this published article.
